# Lymphocyte predominance in blood, pleural fluid, and tumour stroma; a prognostic marker in pleural mesothelioma

**DOI:** 10.1186/s12890-022-01968-2

**Published:** 2022-04-30

**Authors:** Duneesha De Fonseka, David T. Arnold, Anna J. Morley, Mary Brett, Nidhi Bhatt, Anthony Edey, Richard Daly, Anna C. Bibby, Nick A. Maskell

**Affiliations:** 1 Academic Respiratory Unit, University of Bristol, Level 2, Learning and Research Building, Southmead Hospital, Bristol, BS10 5NB UK; 2 Academic Respiratory Unit, North Bristol NHS Trust, Bristol, UK; 3 Cellular Pathology Department, North Bristol NHS Trust, Bristol, UK; 4 Radiology Department, North Bristol NHS Trust, Bristol, UK

**Keywords:** Neutrophils, Lymphocytes, NLR, Pleural fluid, Mesothelioma

## Abstract

**Background:**

As promising novel treatments develop for malignant pleural mesothelioma (MPM), early prognostication has become increasingly important. Circulating and local inflammatory cells are known to play a significant role in other tumour types. We assessed the proportion of lymphocyte populations within blood, pleural fluid and tumour stroma to prognosticate patients with MPM at diagnosis.

**Methods:**

Consecutive patients diagnosed with biopsy-proven MPM were prospectively recruited to an observational cohort study and followed up for a minimum of 7.5 years. Blood and pleural fluid results at presentation were extracted from the medical records. Biopsy specimens were independently reviewed by 2 pathologists who scored the degree of lymphocytic and neutrophilic infiltration.

**Results:**

Baseline results were available for 184 patients. The predominant pleural fluid cell type was calculable for 84 patients and 118 patients had biopsy specimens available for review. A low blood neutrophil/lymphocyte ratio (NLR < 4) inferred a better prognosis with a median survival of 420 days versus 301 days (*p* < 0.01). Survival was better for patients with a lymphocyte-predominant pleural effusion (430 vs 306 days, *p* < 0.01). Lymphocyte infiltration of tumour stroma was also associated with improved survival (n = 92, survival 430 days) compared with neutrophilic or acellular samples (n = 26, survival 342 days *p* < 0.01). In multivariable modelling lymphocyte predominance in blood, pleural fluid and tumour stroma were all associated with a better prognosis.

**Conclusions:**

Lymphocyte predominance within tumour stroma, pleural fluid or blood infers a better prognosis in patients with MPM.

## Introduction

Malignant pleural mesothelioma (MPM) is an aggressive cancer of the pleura with a median survival of 9–14 months from diagnosis. Until relatively recently the mainstay of treatment was palliative chemotherapy (Cisplatin/Pemetrexed) which infers only a modest benefit to survival and quality of life [1, 2]. Recent phase III randomised trials have demonstrated the therapeutic efficacy of bevacizumab and combination immunotherapies, but risk of toxicity makes patient selection critical. Accurate assessment of individual prognosis and predicted disease course is, therefore, highly important [3, 4].

Most malignancies, including MPM, generate local and systemic inflammatory responses, with worse outcomes associated with greater inflammation. Blood inflammatory markers, e.g. total leucocyte count, neutrophil/lymphocyte ratio (NLR), platelet/lymphocyte ratio and C-reactive protein (CRP) have been shown to be inversely correlated with survival in MPM. Infiltration of MPM tumour and stroma by chronic inflammatory cell populations was associated with longer survival in several surgical series, but the studies were confounded by indication for surgery and receipt of chemotherapy (which affects peri-tumour cellular composition) [5, 6].

We assessed the utility of lymphocyte populations in blood, pleural fluid and tumour stroma as baseline prognostic markers in MPM.

## Methods

Data was collected prospectively from consecutive patients diagnosed with MPM at a single centre in the UK, between 03/2008 and 04/2016. Demographics, symptomology, diagnostic method, and treatment received were collected prospectively. Radiological stage at presentation was recorded, according to the International Association for the Study of Lung Cancer (IASLC) classification version 8. All methods were carried out in accordance with ethics guidelines and regulations. Ethical review for the study was waived by the local research and ethics committee (North Somerset and South Bristol Research Ethics Committee then South West 3 REC) who felt that informed consent was not required for analysis of this routine data.

### Variables

The primary outcome was survival, calculated from date of diagnosis until death, with living patients censored on 23/6/21. Explanatory variables under investigation were blood NLR at baseline, pleural fluid cell predominance and tumour stroma cellularity. Known prognostic indicators were included as potential confounders, specifically age (continuous), performance status (0–4; categorical), receipt of chemotherapy (binary) and histological subtype (epithelioid vs non-epithelioid).

### Blood NLR

Blood sampling occurred at initial presentation, prior to receipt of any treatment. The NLR was calculated by dividing the neutrophil count by the lymphocyte count. NLR was handled as a binary variable with a cut-off of ⩾4 (a cut-off used in previous literature [7]).

### Pleural fluid cell predominance

Patients with a pleural effusion underwent diagnostic thoracentesis with fluid sent for cytological analysis. Air dried May Grünwald Giemsa (MGG) stained slides and alcohol fixed Papanicolaou-stained slides were prepared for cytological assessment from the centrifuged deposit of pleural fluid samples. MGG-stained slides were also used for undertaking a semi-quantitative differential cell count. The differential cell count recorded the number of inflammatory cells, mesothelial cells, and macrophages as a percentage of the total number of nucleated cells present in the sample. Cells were counted in multiple areas of each slide to account for variation in distribution. An average value was expressed, giving the relative proportions of inflammatory cells (lymphocytes, neutrophils and eosinophils) and mesothelial cells/macrophages. Cellular predominance was defined according to British Thoracic Society Guidelines, with neutrophil- or lymphocyte-predominant defined as the presence of over 50% of the respective cell type in the absence of ≥ 10% eosinophils, in which case the effusion was deemed eosinophilic. Any effusion not meeting any of the above criteria was classed ‘Mixed’.

### Tumour stroma cellularity

**Fig. 1:**
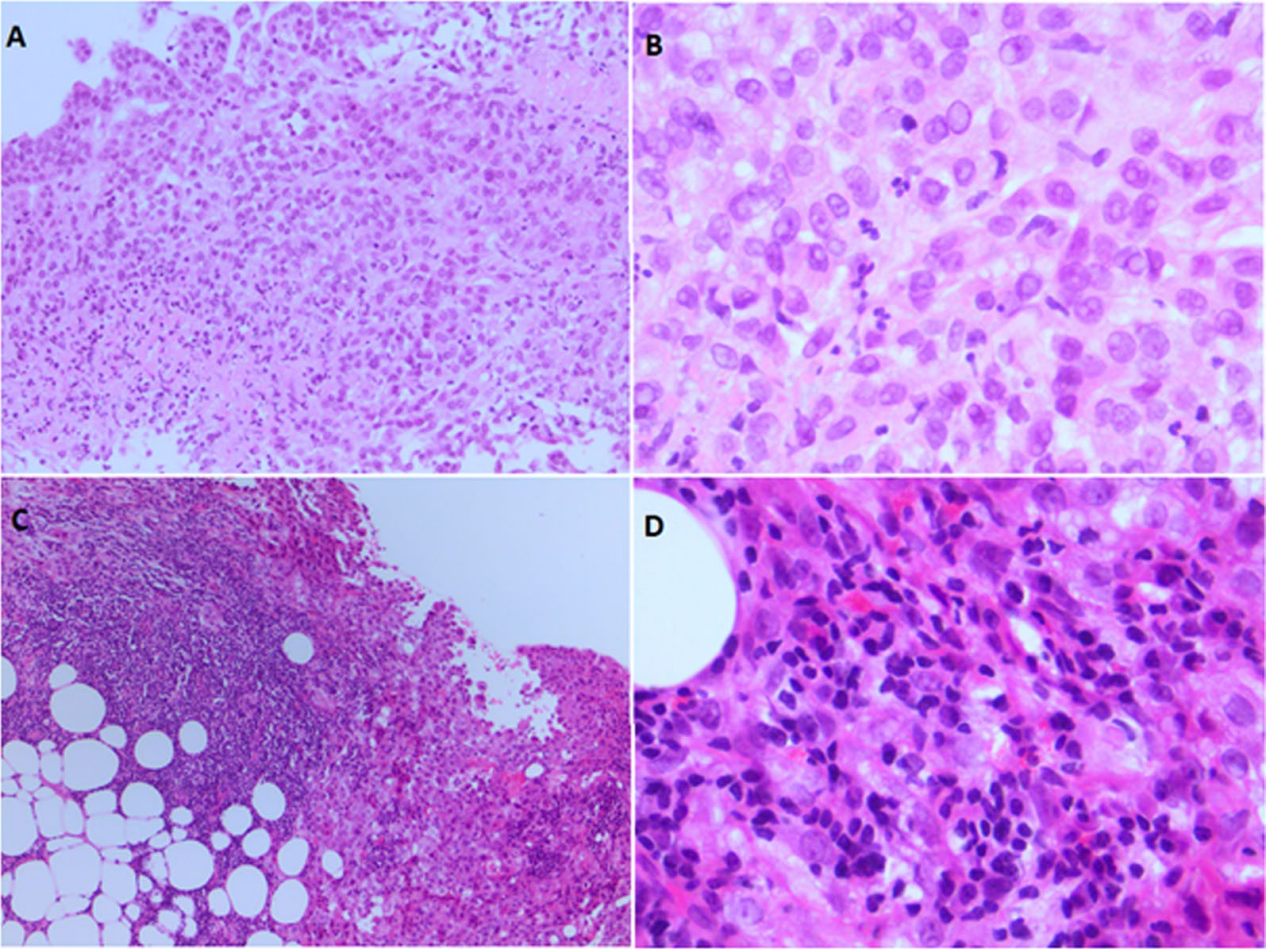
**A**, **B** an epithelioid malignant mesothelioma with neutrophilic stromal infiltration (haematoxylin & eosin stain, original magnification ×100 and ×630). **C**, **D** an epithelioid malignant mesothelioma with lymphocytic stromal infiltration (haematoxylin & eosin stain, original magnification ×100 and ×630)

Pleural biopsies were taken on all patients with a tissue diagnosis of MPM. Biopsy methods were local anaesthetic thoracoscopy, image guided (CT or ultrasound) percutaneous biopsy or video assisted thoracic surgery. Two specialist thoracic histopathologists (NB and RD), independently assessed histology slides. Up to 9 haematoxylin and eosin-stained (H&E) slides were available for each patient and the most representative slides were reviewed. Samples were graded with respect to acute (neutrophilic) and chronic (lymphocytic) inflammation of the tumour stroma surrounding the mesothelioma cells (see Fig. 1). Each slide was graded 0–3 for neutrophilic and lymphocytic infiltration respectively, where 0 indicated an acellular sample and 3 indicated a heavily cellular sample. Biopsy samples were then classified as either neutrophilic or lymphocytic depending on the predominant cell type infiltrating the tumour stroma. If the grading was same for both cell types, the stroma was classified as ‘equal’ and if the grading was ‘0’ for both, the stroma was classified as acellular.

Pathologists were blinded to patients’ baseline characteristics, their radiological staging, blood and pleural fluid markers and long-term outcomes. Where there was initial discordance between pathologists grading, joint assessment was undertaken to reach a consensus.

### Statistical analysis

Statistical analyses were carried out using SPSS V24. Descriptive statistics were used to summarise patients’ characteristics. Overall survival was described and Kaplan Meier survival curves drawn, stratified by each explanatory variable. Differences in survival were compared for each explanatory variable using the log-rank test. Multivariable Cox proportional hazard modelling was carried out to investigate the relationship between explanatory variables and survival, adjusted for all potential confounding variables.

## Results

One hundred and eighty-four patients had histologically proven MPM diagnosed between March 2008 and April 2016. Mean age of our population was 72.4 (range 40–91) years and 160 (87%) of the patients were male, see Table 1 for full baseline information. The majority (n = 90, 49%) received first line chemotherapy with pemetrexed plus cisplatin/carboplatin, the remainder received best supportive care. There were no immunotherapies licensed for MPM at the time this study was recruiting.

**Table 1:** Baseline characteristics of all MPM patients

Baseline characteristics	N = 184
Mean age in years (95% CI)	72.4 (71.2–73.5)
Male	160 (87%)
Stage at diagnosis	
I	60 (33%)
II	8 (4%)
III	73 (40%)
IV	37 (20%)
Missing data	6 (3%)
WHO performance status	
0	49 (27%)
1	85 (46%)
2	23 (13%)
3	8 (4%)
Not documented	19 (10%)
Mode of diagnosis	
LAT	92 (50%)
CT guided	52 (28%)
VATS	31 (17%)
US guided	9 (5%)
Histological subtype	
Epithelioid	120 (65%)
Sarcomatoid	36 (20%)
Biphasic	15 (8%)
Desmoplastic	4 (2%)
Mesothelioma NOS	9 (5%)
Treatment modality	
Best supportive care (only)	90 (49%)
First line chemotherapy (only)	84 (46%)
Second line chemotherapy	10 (5%)
Surgery or radical radiotherapy	0 (0%)

*LAT* local anaesthetic thoracoscopy, *VATS* video assisted thoracic surgery, *NOS* not otherwise specified

Patients were followed up until death, apart from two patients who were alive at the time of analysis, who were censored with follow-up periods of 2708 and 4584 days respectively. Median overall survival was 344 (IQR 171–576 days). Sarcomatoid and desmoplastic subtypes had shorter overall survival at 206 (IQR 102–380) and 128 (IQR 100–302) days, respectively.

### Blood NLR

Median blood NLR was 4.35 (IQR 3.0–6.4). Blood NLR ⩾4 was associated with shorter survival in univariable (HR 0.63, 95% C.I. 0.47–0.84, *p* = 0.002) and multivariable analysis (HR 0.66, 95% C.I. 0.45–0.96, *p* = 0.030).

### Pleural fluid cell predominance

Pleural fluid cell predominance was calculable for 84 patients (45% of the cohort) with the remainder either having no fluid at presentation, incalculable cell differential e.g. due to the presence of blood within the sample, or a cell differential was not performed. Of those evaluated, 43/84 (51%) had a lymphocytic effusion, 9/84 (11%) had a neutrophilic effusion and the remaining 32/84 (38%) were mixed. Patients with a lymphocytic effusion had longer overall survival compared to other cell types (430 vs 306 days). Lymphocyte predominance was positively associated with survival in univariable (HR 0.53, 95% C.I. 0.34–0.82, *p* = 0.005) and multivariable analysis (HR 0.53, 95% C.I. 0.32–0.89, *p* = 0.015). There was no significant correlation between the predominant cell type in pleural fluid and the NLR in blood (*p* = 0.103). A combination of lymphocyte predominance in blood and pleural fluid did not infer improved overall survival with a median survival of 361 days compared to 358 days in the rest of the cohort (*p* = 0.442).

### Tumour stroma cellularity

Of the 184 patients with histologically-confirmed MPM, 118 biopsies were available for examination. Of these, 92/118 (78%) had a predominantly lymphocytic cell infiltration (see Table 2).

**Table 2:** Predominant cell type infiltrating tumour stroma and number of patients in each group

Predominant cell type infiltrating tumour stroma	Number (%)	Survival days (IQR)
Lymphocytic	92 (78%)	368 (198–689)
Neutrophilic	10 (8%)	233 (74–375)
Equal	9 (8%)	204 (108–380)
Acellular	7 (6%)	258 (108–562)

**Fig. 2:**
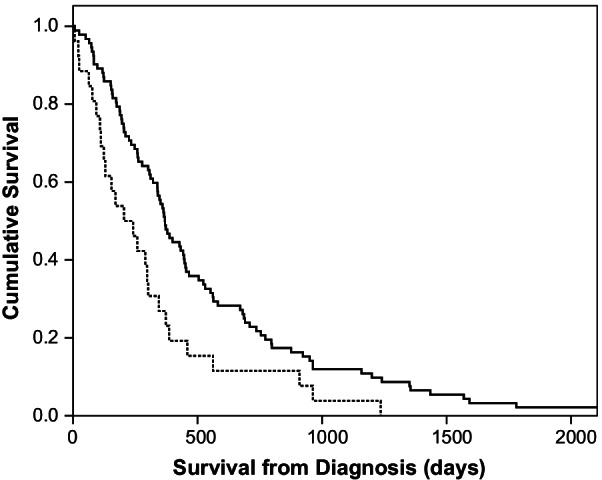
Kaplan–Meir survival curve comparing predominant inflammatory cell type within tumour stroma (solid line; lymphocytic, dashed line; non-lymphocytic)

Survival was longer for patients with lymphocytic infiltration (median 368 days, IQR 197–689)) compared to acellular (n = 7), equal (n = 9) or neutrophilic (n = 10) types (combined median survival of 222 days (IQR 104–375)), see Fig. 2. There was no relationship between the degree of cellular infiltration (on the 1–3 scale) and survival for any cell type. Lymphocytic stroma infiltration was associated with longer survival in univariable (HR 0.74, 95% C.I. 0.60–0.93, *p* = 0.008) and multivariable analysis (HR 0.55, 95% C.I. 0.35–0.87, *p* = 0.010), see Table 3.

**Table 3:** Cox proportional hazards model multivariable analysis for survival

Factor	Univariable HR (95% C.I)	*p*	Multivariable HR (95% C.I)	*p*
Blood NLR (< 4)	0.63 (0.47–0.84)	0.01	0.66 (0.45–0.96)	**0.03**
Pleural fluid lymphocyte predominance	0.52 (0.33–0.82)	0.01	0.53 (0.32–0.89)	**0.02**
Lymphocytic stroma predominance	0.74 (0.59–0.93)	0.01	0.55 (0.35–0.87)	**0.01**
Age (per year)	1.02 (1.01–1.04)	0.01	0.98 (0.95–1.01)	0.15
Male gender	0.85 (0.56–1.29)	0.45	0.66 (0.40–1.10)	0.11
WHO PS (3–4)	1.37 (1.12–1.67)	0.01	2.60 (1.72–3.92)	**0.01**
Epithelioid sub type	0.61 (0.44–0.846)	0.01	0.60 (0.39–0.93)	**0.02**
Radiological Stage (1 & 2 vs 3 & 4)	0.95 (0.53–1.69)	0.08	0.74 (0.32–1.69)	0.47
Received chemotherapy	0.52(0.40–0.70)	0.01	0.58 (0.40–0.84)	**0.04**

*NLR* neutrophil/lymphocyte ratio, *p* < 0.05 defined as significant

### Other prognostic factors

In the multivariable survival model, other independent prognostic markers were higher WHO performance status (HR 2.60; 95% C.I. 1.72–3.93, *p* = 0.001), epithelioid histological subtype (HR 0.60; 95% C.I. 0.39–0.93, *p* = 0.022) and receipt of chemotherapy (HR 0.58; 95% C.I. 0.40–0.84, *p* = 0.004, see Table 3.

## Discussion

MPM has a highly variable disease course that is to predict at diagnosis. This study investigates the prognostic role of the predominant inflammatory cell type within routinely collected diagnostic samples. Using a prospectively collected cohort it demonstrates that a lymphocyte predominance in pleural fluid and tumour, as well as low blood NLR, infers a better overall survival.

Previous studies have investigated inflammatory markers as prognostic markers in MPM, including NLR, CRP, platelet/lymphocyte ratio, total leucocyte count and total lymphocyte count [8]. Our results for blood neutrophil–lymphocyte ratio validate prior findings. A study by Kao et al. with 173 patients receiving chemotherapy found a median survival of 16.7 months for those with NLR < 5 at baseline, compared to 6.6 months for those with an NLR > 5 [9]. In our study, we observed similar median survival of 421 days (15.1 months) for NLR < 5 versus 240 days 3(8.6 months) for NLR > 5 (*p* < 0.01). A study by Hooper et al. found a significant difference in survival with 453 versus 257 days for those with NLR < 4 compared to NLR ⩾ 4 [7].

To our knowledge, this is the first study to investigate the significance of tumoural inflammatory cell infiltration in a cohort of patients not undergoing radical surgery for MPM, and who had not yet received any systemic treatment. We demonstrated a significant difference in survival between patients with a lymphocytic cell infiltration around the tumour cells, suggesting a more chronic inflammatory response. Suzuki and colleagues reported a cohort of 175 patients who had undergone radical surgery for epithelioid MPM [5]. They investigated the role of acute and chronic inflammatory response and level of inflammatory cell infiltration in excised mesothelioma tumour and stroma. They showed that patients with a lymphocyte predominance in the tumour stroma had improved survival (median OS = 19.4 vs. 15.0 months, *p* = 0.01). Suzuki et al. did not show a difference in survival between high and low-grade infiltration by inflammatory cells, and a post-hoc analysis of our data did not either. Other smaller series have identified similar patterns within tumour, with higher levels of CD8 (+) tumour-infiltrating lymphocytes associated with better prognosis in post-surgical patients [6]. There are two potentially overlapping hypotheses for this finding. Firstly, that lymphocytes (e.g. CD8 T-cells) are playing a role in the response to the tumour suppressing its growth and angiogenesis. Secondly that an abundance of lymphocytes simply reflects chronicity of naturally more indolent tumour subtypes at the point of biopsy.

Although a number of studies have looked at pleural fluid biochemical parameters for prognostication of malignancy, few have investigated pleural fluid lymphocyte predominance in MPM. A recent study by Popowicz and colleagues found that in malignant effusions of all types (n = 117) a higher ratio of lymphocytes to neutrophils was associated with improved survival, although the study protocol did not specify that the sample needed to be collected at the time of diagnosis [10]. In our study we found that where the pleural fluid predominance was calculable this was an independent prognostic marker. However, given that not all patients present with pleural effusions that can be sampled and that cytological assessment can be limited by the presence of blood or poor cellular quality, this finding was applicable to a minority of patients (45%).

Our study is not without limitations. This is a single centre study conducted in the pre-immunotherapy era, although samples were collected from consecutive patients prior to chemotherapy reducing confounding by treatment. Consequently, the results may not be relevant to people receiving immune checkpoint inhibition. Secondly, only 118 biopsy samples out of our original 184 patient cohort were available for analysis. Finally, biopsy samples were generally small samples, as is typical for diagnostic samples from CT guided biopsy or medical thoracoscopies, compared with some of the other studies using larger surgical samples. Given the heterogeneous nature of MPM tumours, smaller biopsies may not provide an accurate representation of the entire tumour. However, we have shown that even the smaller samples obtained without surgery can provide crucial information that could influence the management of patients with MPM.

## Conclusions

In conclusion, we demonstrate that inflammatory cells in the systemic circulation and local environment of MPM can offer prognostic information that is valuable to both physician and patient. Lymphocyte predominance in blood, pleural fluid and tumour stroma are independent markers of improved overall survival.

## Data Availability

All data generated or analysed during this study are included in this published article.

## Abbreviations

C.I: Confidence interval
CRP: C reactive protein
CT: Computed tomography
H&E: Haematoxylin and eosin-stained
HR: Hazard ratio
IASLC: International Association for the Study of Lung Cancer
IQR: Interquartile range
MGG: May Grünwald Giemsa
MPM: Malignant pleural mesothelioma
NLR: Neutrophil lymphocyte ratio
